# Minimum 3.5-year outcomes of operative treatment for Achilles tendon partial tears in the midportion and retrocalcaneal area

**DOI:** 10.1186/s13018-020-01856-7

**Published:** 2020-09-10

**Authors:** Heinz Lohrer

**Affiliations:** 1 European SportsCare Network (ESN), Zentrum für Sportorthopädie, Borsigstrasse 2, 65205 Wiesbaden-Nordenstadt, Germany; 2 Department for Sports and Sport Science, Albert-Ludwigs-Universität Freiburg i. Brsg., Schwarzwaldstraße 175, 79117 Freiburg, Germany

**Keywords:** Achilles tendon partial tear, VISA-A, Haglund’s disease, Retrocalcaneal bursitis, Operation, Results

## Abstract

**Background:**

Achilles tendon partial tears are not easy to diagnose and to manage. Most frequently, they are located in the midportion and insertional area. These entities result from different pathologic pathways, and different treatment strategies are applied. The outcome is rarely investigated.

**Methods:**

This study includes patients who underwent surgery for partial tears in the midportion or retrocalcaneal Achilles tendon area between the years 2009 and 2015 by a single surgeon. Patients were prospectively assessed preoperatively and 3, 6, and 12 months postoperatively, using the VISA-A-G questionnaire. The final retrospective follow-up was performed after a minimum of 3.5 years postoperatively. Forty-eight Achilles tendon partial tears at the level of the retrocalcaneal bursa (impingement lesions) and 27 midportion Achilles tendon partial tears were identified. After applying rigorous exclusion criteria, 21 and 16 cases, respectively, remained for the final follow-up. Results were analysed by inferential and descriptive statistics.

**Results:**

The VISA-A-G outcome scores improved significantly from preoperative to 6 months, 12 months, and final postoperative assessment. Preoperatively, the average VISA-A-G score was 42.1 (range, 18–73) for patients operated for Achilles tendon partial tears at the level of the retrocalcaneal bursa and 44.6 (range, 10–73) for the midportion Achilles tendon partial tear group, respectively. At final follow-up 88.8 (range, 15 to 100) and 96.9 (range, 71 to 100) were scored in the respective treatment groups. A repeated measures ANOVA determined that mean performance levels showed a statistically significant difference between measurements (*p* < 0.001). There was no systematic effect found between groups (*p* = 0.836).

**Conclusions:**

In Achilles tendon partial tears recalcitrant to conservative treatment, operative intervention is highly successful in most cases, irrespective of the level of the injury. Results were statistically equal when comparing the midportion and retrocalcaneal Achilles tendon partial tear groups.

**Trial registration:**

DRKS, DRKS00014266. Registered 06 April 2018. ‘Retrospectively registered’, https://www.drks.de/drks_web/navigate.do?navigationId=results.

## Background

‘Subcutaneous partial rupture of the Achilles tendon’ was first described in 1968 [1]. The author presented 24 cases and defined that entity as a ‘tear involving a varying number of fibres in the free portion of the Achilles tendon, usually leaving most fibres intact’ [1]. So far, only little research has been conducted. In a PubMed/Medline internet search (20 March 2020), 79 articles were found for [Achilles tendon] and [partial] and [(tear) or (rupture)]. Twenty-one papers described imaging. There were 10 animal studies and two anatomic descriptions. Four papers presented overviews without original data. Thirteen studies focused on Achilles tendinopathy and nine on Achilles tendon ruptures. Eight publications were not relevant for the topic. Twelve clinical original case series and case studies presented 213 cases. From these, 83 lesions affected the midportion Achilles tendon, while 130 were located in the retrocalcaneal Achilles tendon area.

Clinical presentation of Achilles tendon partial tears is unspecific in most cases and is frequently not different from Achilles tendinopathy [1–3] or retrocalcaneal bursitis [4]. Suspicion of Achilles tendon partial tear is likely, when the patient experiences an acute onset, an audible pop, and a piercing pain during load [3, 5]. Dependent on the size and the age of the lesion, physical examination inconsistently presents Achilles tendon swelling or denting and calf muscle atrophy. Side differences in ankle dorsiflexion indicate Achilles tendon elongation in an advanced stage [6].

Diagnostic ultrasound imaging, colour Doppler, and/or MRI can underline the clinical suspicion [3, 7–9]. Ultrasound and power Doppler investigations demonstrated unspecific findings like localised swelling, reduced echogenicity, and neovascularisation related to the injured area [3, 7]. More specific but inconsistent findings were discontinuity of tendon fibres and intratendineous anechogenic or low echogenic spots [3]. However, ‘especially partial ruptures of the Achilles tendon’ are not sufficiently detected by ultrasound [10]. MRI scans have the highest accuracy for Achilles tendon partial tears [9, 11].

Conservative treatment should initially be initiated and contain most modalities used also for Achilles tendinopathy [12, 13]. However, caution against eccentric training is recommended, as it may increase the risk for total Achilles tendon rupture [2]. The reviewed literature presents only six (four midportion, two impingement) partial Achilles tendon tears with successful conservative therapy. When unresponsive to conservative therapy, operative procedures are recommended [1].

In the midportion area, Achilles tendon partial tears are operatively addressed by excision and side-to-side and/or end-to-end repair [1, 2, 4]. In the retrocalcaneal area, the bursa and the Haglund tuberosity are removed open or endoscopically. Some authors additionally repair this so-called impingement partial tear [4, 14, 15] while others only excise the lesion [16, 17].

In a previous study, we compared results of operative treatment for Achilles tendinopathy and retrocalcaneal bursitis with or without Achilles tendon partial tears [4]. That study demonstrated no difference in outcome between the four respective groups, but the available numbers and the resulting power of that study were small.

The aim of this study was to compare the patient-related outcome of operatively treated Achilles tendon partial tears when located in the midportion or in the retrocalcaneal area after a minimum of 42 months and at 3, 6, and 12 months. Besides, the VISA-A-G (Victorian Institute of Sports Assessment–Achilles tendon, German version) outcomes within the groups were compared from preoperative to 3, 6, 12, and more than 42 months postoperative.

## Material and methods

### Ethics

The Landesärztekammer Hessen Ethics Committee (FF 162/2016) approved this study.

Informed consent was obtained from all patients, and the rights of the patients were protected. The registration trial number is DRKS00014266 on DRKS. ‘Retrospectively registered’. Date of registration: 06 April 2018.

### Patients and grouping

**Fig. 1:**
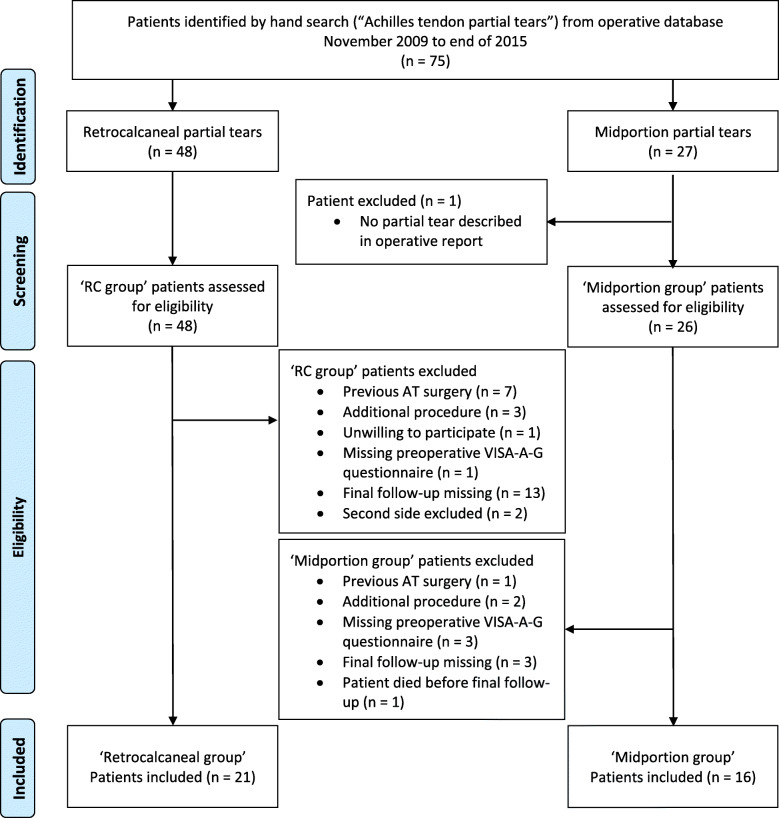
Flow chart of group division and the selection of analysed patients for the study. Adapted from Moher et al. [18]. VISA-A-G, Victorian Institute of Sport Assessment-Achilles tendon-German version

The patients of this study were operated between November 2009 and end of 2015. We searched our electronic databases for patients operated for Achilles tendon partial tear. Dependent on the anatomic level of the Achilles tendon partial tear and from the respective operative procedure, we enrolled patients either to a ‘midportion group’ or to a ‘retrocalcaneal group’ (Fig. 1 [18]). To be included, patients preoperatively had to be unresponsive to two or more of the following conservative treatment modalities: load modification, rest, acupuncture, orthotics, bandage, NSAID, eccentric exercises, physiotherapy, ice, ESWT, injections, and radiation. The analyses comprised only datasets of patients, who responded to the final follow-up questionnaire. Two patients included, both from the ‘retrocalcaneal group’, underwent bilateral operations within 3 weeks and 2 months. The respective final follow-up VISA-A-G scores for these four lower extremities were 100, 100, 100, and 88. To avoid ‘double dipping’ effects [19] only the scores obtained from the sides operated on first were included (100 and 88).

Further exclusion criteria were no partial tear described in the operative report (*n* = 1), previous Achilles tendon surgery (*n* = 8), additional procedures performed during surgery (*n* = 5), unwilling to participate (*n* = 1), missing preoperative VISA-A-G questionnaire (*n* = 4), final follow-up missing (*n* = 16), and second side excluded (*n* = 2). One patient died between the 12 months and the final follow-up for reasons not related to the Achilles tendon surgery. Three midportion and three retrocalcaneal Achilles tendon partial tear patients (operated between 2009 and 2011) were already enrolled in a previous evaluation [4].

### Diagnostics

History was nonspecific in most cases and was not different from Achilles tendinopathy or chronic retrocalcaneal bursitis. Patients generally complained about sport/running-induced pain in the involved Achilles tendon. This pain increased over time and increasingly limited the patients’ activity. In typical cases, an acute event exacerbated the symptoms (Table 1).

**Table 1:** Details of the preoperative history of the included patients

	Patient no.	Preoperative sport	Injury mechanism	Concomitant conditions	Prodromal symptoms (months)	Previous conservative therapy	MRI	Diagnostic delay [months]	Preop. pain while walking	Running impossible since (months.)
Impingement partial tear	1	Marathon	Spontaneous	Left knee	36	Cortisone injections, physiotherapy, orthotics, radiotherapy, ESWT, NSAID	Longitudinal partial tear	18	Yes	14
	2	Tennis, jogging	Cutting	No	132	ESWT, cortisone injections, physiotherapy, orthotics, radiotherapy, thermocoagulation, rest	Longitudinal partial tear	132	Yes	12
	5	No	Spontaneous	Left ACL reconstruction (5 years ago), right AT rupture (12 years ago), hyperuricemia, Hashimoto thyreoiditis, blood group 0	48	N.s.	N.s.	48	Yes	N.a.
	6	Ballet	Spontaneous	left hip endoprosthesis	6	N.s.	Retrocalcaneal bursitis, insertional tendinopathy	6	Yes	0
	8	Tennis, jogging	Spontaneous	No	25	Cortisone injections, physiotherapy, sport reduction	N.p.	25	Yes	0
	11	Fitness	Hopping	No	26	Orthotics, viscoelastic heel lift, sport reduction/rest, cortisone injections	Retrocalcaneal bursitis	6	Yes	6
	12	Jogging	Spontaneous	No	30	Physiotherapy, stretching, ESWT	Retrocalcaneal bursitis	7	Yes	N.s.
	13	Jogging	Spontaneous	No	96	Cortisone injections	N.p.	N.s.	Yes	0
	14	Marathon	Spontaneous	No	19	N.s.	Partial tear	4	Yes	0
	15	Marathon	Squash	AT rupture left side	18	Cortisone injection, ice, ESWT, physiotherapy, cream	N.s.	12	Yes	No
	18	Marathon	Spontaneous	Surgery for left RB 3 weeks later	15	Orthotics, physiotherapy, reduced running, massage, heel lift, cortisone injections	N.p.	15	No	0
	20	Squash	Spontaneous	No	180	Orthotics, physiotherapy, reduced sport, cortisone injections	N.p.	180	No	0
	21	Jogging	Spontaneous	Achilles tendinopathy contralateral side	24	N.s.	N.p.	24	Yes	N.s.
	23	Tennis, jogging	Spontaneous	RB contralateral side	24	Cortisone injections, ESWT	N.p.	24	Yes	6
	25	Jogging	Running	No	6	Cortisone injections, ESWT	Partial tear	6	Yes	6
	27	Dancing	Spontaneous	No	96	N.s.	Partial tear	N.s.	Yes	N.s.
	28	400 m hurdles	Spontaneous	No	17	ESWT, physiotherapy, ozone therapy	Partial tear	0	No	No
	30	Marathon	Spontaneous	No	24	Cortisone injections	N.p.	24	N.s.	N.s.
	31	Tennis, jogging	Spontaneous	Osteochondral lesion med. talus	25	ESWT, acupuncture	Retrocalcaneal bursitis	25	No	No
	33	Marathon	Spontaneous	AT right side	60	Physiotherapy, laser, acupuncture, Orthotics, ESWT, cortisone injection	N.p.	N.s.	Yes	No
	34	Ju-Jutsu	Spontaneous	AT partial tear left (27 years), right (7 years) ago, conservative	36	Physiotherapy, cortisone injections	Retrocalcaneal bursitis	36	Yes	N.s.
		**Mean**			**44.9**			**32.9**		**4**
		SD			43.8			45.8		5.0
		**Median**			**25**			**21**		**0**
		Maximum			180			180		14
		Minimum			6			0		0
Midportion partial tear	1	Volleyball	Domestic fall	No	11	Physiotherapy	Longitudinal partial tear	6	Yes	N.a.
	2	Jogging	Spontaneous	CAI	96	Physiotherapy, stretching, rest, ESWT, orthotics	Partial tear posterior column	3	Yes	3
	3	Marathon	Spontaneous	Ipsilat. peroneus lg. tendon surgery 12 years ago, ipsilat AT partial tear 11 years ago	5	N.s.	Partial tear posterior column	10	No	No
	6	Jogging	Spontaneous	No	5	NSAID, radiation, physiotherapy	Longitudinal partial tear	4	Yes	N.s.
	7	Marathon	Spontaneous	M. Meulengracht	132	Injection (substance n.s.)	Partial tear	1	Yes	11
	8	Tennis	3 tear-like events	No	12	Physiotherapy, radiotherapy, training reduction, orthotics, ESWT, eccentrics	N.p.	12	Yes	6
	9	No	2 tear-like events (6 and 0.5 months ago)	Pheochromocytoma, Hypercholesterolemia, Hypertriglyceridemia, PCL rupture contralateral	18	ESWT, acupuncture, 3 cortisone injections, stretching, orthotics, chiropractic	Partial tear anterior	0	Yes	N.s.
	10	Football (professional)	Tear-like event (2 months ago) during football practise	No	2	Physiotherapy, cortisone injection, eccentrics	‘Small’ partial tear	2	No	2
	11	Basketball	Step over a kubstone	No	36	Injection (substance n.s.)	Partial tear posterior column	0	Yes	12
	12	Football	Spontaneous	No	120	Injection (substance n.s.)	Cystic posteromedial column	6	Yes	6
	14	Triathlon	Tear-like event (3 months ago) during running	No	120	Rest, physiotherapy	Partial tear medial column	N.s.	Yes	3
	15	Marathon	Tear-like event (5 months. ago) during running	S1 lesion (26% isokinetic calf muscle deficit)	98	Load reduction, glucose injections, orthotics, physiotherapy, eccentrics, ESWT, ice	N.p.	0,5	Yes	4,5
	16	Marathon	Spontaneous	Medial Gonarthrosis and posterior instability opposite side, giant cell tumour cuboid	60	Eccentrics	N.p.	N.s.	Yes	3
	18	Tennis	Spontaneous	M. Meulengracht	15	Load reduction	N.p.	11	Yes	15
	19	Tennis	Spontaneous	No	9	ESWT, eccentrics, physiotherapy, orthotics	N.p.	9	Yes	9
	20	Football	Spontaneous	No	15	Cortisone injections, physiotherapy	Partial tear dorsal column central	15	Yes	5
		**Mean**			**47.1**			**5.7**		**6.6**
		SD			47.2			4.8		4.0
		**Median**			**16.5**			**5**		**5.5**
		Maximum			132			15		15
		Minimum			2			0		2
P impingement vs. midportion partial tear group					0.203			**0.001**		0.078

Significant results are displayed in bold

*SD* standard deviation, *n.s.* not specified, *n.p.* not performed, *med*. medial, *ipsilat*. ipsilateral, *lg*. longus, *M*. Morbus, *PCL *posterior cruciate ligament, *AT *Achilles tendon, *RB *retrocalcaneal bursitis, *CAI *chronic ankle impingement

Physical examination allocated the patient’s symptoms to the injured Achilles tendon region. The most important finding was the localised pain on palpation related either to the Achilles tendon midportion or to the retrocalcaneal area. Midportion lesions additionally demonstrated a spindle-shaped swelling at the Achilles tendon 2–7 cm above its calcaneal insertion. Retrocalcaneal lesions presented with swelling related to the Haglund/retrocalcaneal region.

All patients underwent ultrasound and power Doppler investigations, and MRI scans were available for 21 patients (Table 1).

### Operative procedures

A single orthopaedic surgeon performed all procedures. The operative techniques for the two different lesions have already been described in detail [4]. Midportion Achilles tendon partial tears were accessed by transverse or longitudinal skin incisions. The paratenon was resected, and the anterior Achilles tendon was released. Following longitudinal splitting of the Achilles tendon, the lesion was identified and excised. Repair comprised transverse anterior O-shaped side-to-side and a posterior running suture (2-0 and 3-0 Vicryl). In five cases, a plantaris tendon transplant reinforced the reconstruction. For retrocalcaneal Achilles tendon partial tears, an oblique to transverse or a longitudinal skin incision at the lateral Achilles tendon border was made over the lateral aspect of the retrocalcaneal bursa. At the lateral Achilles tendon border, the retrocalcaneal bursa was accessed by a longitudinal incision. The subcutaneous bursa, the retrocalcaneal bursa, and Haglund’s tuberosity were removed from lateral to medial. By elevating the lateral Achilles tendon border, the anterior Achilles tendon partial tear was identified and debrided, and the lesion was repaired with one to five Z- or O-shaped transverse 2-0 Vicryl sutures [15].

### Postoperative care

Postoperative treatment and rehabilitation regimen was not different for the two groups. Postoperatively, a Scotchcast splint was applied for 3–4 days and remained for 4 weeks postoperatively as a night splint. A stable shoe with a 1.5–2-cm heel lift was initiated 3–4 days postoperatively during the day, and load was gradually increased during the following 1–2 days. About 6 weeks postoperatively, the heel lift was reduced to 1 cm. The stable shoe was discontinued 6 weeks postoperatively but the 1-cm heel lift remained in the patient’s normal shoe for six more weeks. After the 12th postoperative week, the patient, if free of pain, was allowed to gradually commence running activities. If symptom free, full load in practice and during competitions were allowed 6 months postoperatively.

### Follow-up

There is no validated research tool available for Achilles tendon partial tears. Therefore, outcome was prospectively evaluated using the VISA-A-G questionnaire preoperatively (baseline) and 3, 6, and 12 months and after a minimum of 3.5 years postoperatively. The VISA-A questionnaire is the only valid, reliable, and disease-specific patient-administered questionnaire for research in Achilles tendinopathy [20–22]. It measures the severity of pain and function, related to activities of daily living (six items) and during sport (two items). A score of 0 means a maximal impairment and 100 reflects an asymptomatic person. In principle, the VISA-A questionnaire is designed and validated only for Achilles tendinopathy in the midportion and/or retrocalcaneal area [20–22]. However, the VISA-A score correlates with the ATRS (Achilles tendon Total Rupture Score) [23]. Both tools are not specifically validated for Achilles tendon partial tears, and the ATRS is not available in German language. We therefore decided to use the VISA-A-G questionnaire as the best suitable tool for this study. Two patients (one in each group) were not involved in any sport. Corresponding to the proposed procedure [24], their results were calculated from the percentage result of questions 1 to 6 only.

Two patients in the retrocalcaneal group underwent bilateral operations (3 weeks and 2 months interval). For these patients, only the results for the side of the initial intervention were further analysed.

A VISA-A-G score of 90 and more was regarded as excellent, 70–89 as good, and below 70 as unsuccessful [25]. From this classification, success rate is defined as the summarised excellent and good results [4, 26].

The clinical records were retrospectively screened for the clinical status, including ultrasound and MRI (if applicable). The clinical appointments were conducted according to the individual rehabilitation process of the patients. A specific clinical and imaging follow-up was not scheduled. Power Doppler ultrasound results were graded according to previous research [27].

### Statistical analysis

Inferential statistical analysis was carried out using SPSS 26.0 (IBM Inc., USA). Repeated measures one way ANOVA was conducted for the VISA-A-G score to identify between group differences, time effects, and group x time interaction effects. For 9% missing values, the last observation carried forward technique has been applied. Further evaluated parameters (anthropometry, pre- and postoperative clinical data) were compared descriptively between groups or, if applicable, analysis using the unpaired *T* test for normally distributed parameters or the Mann-Whitney *U* test for non-normally distributed values (extent of resection, initiation of running activities postoperatively). The significance level was set at *p* = 0.05. Results are reported as means with corresponding standard deviations (SD) and ranges.

## Results

### VISA-A-G outcome

There was homogeneity of covariances, as assessed by Box’s test (*p* = 0.053). Mauchly’s test of sphericity indicated that the assumption of sphericity had not been violated, *χ*^2^(9) = 12.042, *p* = 0.211. A repeated measures ANOVA determined that mean performance levels showed a statistically significant difference between measurements, F (4, 140) = 73.40, *p* < 0.001, partial *η*^2^ = 0.68 (Tables 2, 3, and 4). There was no significant main effect for group, F (1, 35) = 1.979, *p* < 0.168, partial *η*^2^ = 0.054. There was no statistically significant interaction between time and group, F (4, 140) = 0.361, *p* = 0.836 (Table 2).

**Table 2:** Results of the operative reports’ evaluation, postoperative chart analyses, and pre- and postoperative VISA-A-G questionnaire scores for the included patients

	Patient No.	Tear orientation	Operative technique	Number of transverse sutures	Extent of resection (%)	Complications	Running activity initiated (months postop.)	VISA-A-G Score					Final follow-up (months)
								Preop.	3 months postop.	6 months postop.	12 months postop.	Final follow-up	
Impingement partial tear	1	Transversal + longitudinal	RCB + H + debridement	3 ant. Z	10	Reinjury 9 weeks postop. (walking at the beach). MRI = 20% partial tear, reoperation proposed	n.p.	33	12	8	22	15	42
	2	Longitudinal	RCB + H + debridement	3 ant. Z	10	No	13	28	32	23	m.v.	58	50
	5	Transversal + longitudinal	RCB + H + debridement	2 trans. Z	10	No	N.a.	56	53	70	94	93	50
	6	Transversal + longitudinal	RCB + H + debridement	5 ant. Z	20	No	5	18	56	80	95	97	51
	8	Transversal + longitudinal	RCB + H + debridement	3 ant. Z	10	No	5	31	45	81	99	88	51
	11	Longitudinal medial	RCB + H + debridement	2 ant. Z	5	DVT	N.s.	41	28	79	67	87	56
	12	Transversal + longitudinal	RCB + H + debridement	3 ant. O	10	Midportion AT partial tear 3 months postop.	10	28	37	93	54	82	57
	13	Longitudinal	RCB + H + debridement	2 ant. Z	5	No	2	53	58	75	84	99	59
	14	Transversal	RCB + H + debridement	2 ant. O	15	No	7	26	m.v.	m.v.	96	96	63
	15	Transversal + longitudinal	RCB + H + debridement	2 ant. O	20	No	3	55	61	97	97	97	65
	18	Transversal + longitudinal	RCB + H + debridement	3 ant. O, 1 trans. O	20	No	4	48	81	80	94	97	71
	20	Transversal + longitudinal	RCB + H + debridement	3 ant. O	10	No	5	23	20	92	100	100	74
	21	Longitudinal	RCB + H + debridement	2 trans. O, 1 ant. O	15	No	3	43	52	m.v.	95	96	78
	23	Transversal + longitudinal	RCB + H + debridement	2 trans. O	20	N.s.	N.s.	42	62	98	100	100	77
	25	Transversal + longitudinal	RCB + H + debridement	1 ant. Z	10	No	4	48	34	87	100	89	79
	27	Longitudinal	RCB + H + debridement	3 trans. O	15	N.s.	N.s.	50	m.v.	m.v.	m.v.	100	90
	28	Longitudinal	RCB + H + debridement	3 trans. O	10	No	3	40	m.v.	m.v.	67	83	111
	30	Longitudinal	RCB + H + debridement	2 ant. O	10	No	6	73	49	68	93	93	114
	31	Longitudinal posterior, transversal anterior	RCB + H + debridement	4 trans. O	25	No	5	50	50	95	96	98	107
	33	Transversal	RCB + H + debridement	4 trans. O	40	No	4	51	51	91	90	94	107
	34	Transversal	RCB + H + debridement	2 ant. O	10	DVT 12 days postop.	4	48	78	94	94	97	107
				**Mean**	**14.3**		**5.2**	**42.1**	**47.7**	**77.1**	**86.2**	**88.8**	**74.2**
				SD	7.8		2.7	13.0	17.7	24.4	19.7	19.0	22.7
				**Median**	**10.0**		**4.5**	**43.0**	**50.5**	**81.0**	**94.0**	**96.0**	**71.0**
				Max.	40		13	73	81	98	100	100	114
				Min.	5		2	18	12	8	22	15	42
Midportion partial tear	1	Longitudinal dorsal	Debridement	4 ant. trans. O	20	Focal wound healing disorder (7 mm)	N.s.	59	83	91	m.v.	94	44
	2	Longitudinal dorsal medial	Debridement	4 ant. trans. O	15	No	4	68	68	94	94	100	42
	3	Longitudinal 8 cm	Debridement	3 ant. trans. O	10	No	4	73	36	88	97	100	57
	6	Longitudinal 8 cm	Debridement	6 ant. trans. O	40	2 months postop. sero-hematoma (3 ml punctured, 2 PRP injections)	N.s.	17	56	96	96	100	70
	7	Longitudinal 10 cm	Plantaris	3 ant. trans. O	60	Initial redness, 1 week postop, oral clindamycin	5	63	83	93	97	100	64
	8	Longitudinal dorsal medial	Plantaris	3 ant. trans. O	50	11 months later Haglund resection ipsilateral	5	38	51	58	m.v.	100	68
	9	Transverse anterior and longitudinal dorsal proximal	Plantaris	5 ant. trans. O	50	No	N.s.	20	38	m.v.	m.v.	100	87
	10	Transverse anteromedial 1.5 mm	Debridement	3 ant. trans. O	10	No	3	43	33	95	97	100	89
	11	Cystic sero-hematoma, longitudinal	Debridement	4 ant. trans. O	35	No	N.s.	61	44	64	96	71	87
	12	Longitudinal 4.5 cm	Debridement	3 ant. trans. O	10	No	3.5	49	m.v.	89	100	100	91
	14	Longitudinal medial 3 cm	Debridement	2 ant. trans. O	5	No	3.5	63	84	m.v.	97	100	104
	15	Longitudinal lateral	Debridement	2 ant. trans. O	5	No	5	10	61	52	80	94	97
	16	Longitudinal medial 4.5 cm	Debridement	3 ant. trans. O	5	No	7	66	50	61	75	97	107
	18	Transverse anteromedial	Plantaris	5 ant. trans. O	50	No	6	21	49	m.v.	100	100	103
	19	Transverse anteromedial	Debridement	2 ant. trans. O	15	DVT 16 days postop.	3.5	48	68	m.v.	97	94	107
	20	Longitudinal 8 cm	Debridement	5 ant. trans. O	15	No	6	15	56	91	100	100	114
				**Mean**	**24.7**		**4.6**	**44.6**	**57.3**	**81.0**	**94.3**	**96.9**	**83.2**
				SD	18.7		1.2	21.0	16.4	16.1	7.4	7.1	22.2
				**Median**	**15.0**		**4.5**	**48.5**	**56.0**	**90.0**	**97.0**	**100.0**	**88.0**
				Max.	60		7	73	84	96	100	100	114
				Min.	5		3	10	33	52	75	71	42

Significant results are displayed in bold

*RCB* retrocalcaneal bursa, *H* Haglund, *ant.* anterior, *trans*. transverse, *n.s.* not specified, *postop.* postoperative, *m.v.* missing value, *n.p.* not possible, *MRI *magnetic resonance imaging, *SD *standard deviation, *Max*. maximum value, *Min*. minimum value, *DVT *deep vein thrombosis, *mm *millimeter, *ml *milliliter

**Table 3:** Longitudinal ANOVA comparison of the VISA-A-G results for the retrocalcaneal partial tear group

	3 months postop.	6 months postop.	12 months postop.	Final follow-up
Preoperative	1.000	**0.001**	**< 0.001**	**< 0.001**
3 months postop.		**0.001**	**< 0.001**	**< 0.001**
6 months postop.			0.281	**0.004**
12 months postop.				0.368

Significant results are displayed in bold

*Postop*. postoperative

**Table 4:** Longitudinal ANOVA comparison of the VISA-A-G results for the midportion partial tear group

	3 months postop.	6 months postop.	12 months postop.	Final follow-up
Preoperative	0.589	**0.001**	**< 0.001**	**< 0.001**
3 months postop.		**0.029**	**0.042**	**< 0.001**
6 months postop.			**0.005**	**0.005**
12 months postop.				1.000

### Success rates

At final follow-up, excellent results/full recoveries in the retrocalcaneal partial tear and the midportion partial tear group were found in 14/21 (67%) and 15/16 (94%) cases, respectively. Good results were found in 5/21 (24%) and 1/16 (6%) patients in the retrocalcaneal partial tear and the midportion partial tear group, respectively. Unsuccessful outcome at final follow-up was found in 2/21 (9.5%) patient in the retrocalcaneal partial tear group. The success rate is 91% for retrocalcaneal partial tear and 100% for the midportion partial tear group.

### Anthropometric data

There were 11 males and 10 females within the retrocalcaneal partial tear group, while the midportion partial tear group comprised 15 males and 1 female. Patients’ age at surgery in the retrocalcaneal partial tear and in the midportion partial tear group was 51 ± 9.2 (range, 20–65) and 50 ± 9.3 (range, 23–66) years (*p* = 0.724), respectively. Patients’ height in the retrocalcaneal partial tear and in the midportion partial tear group was 176 ± 9.4 (range, 158–192) and 183 ± 6.1 (range, 174–193) cm (*p* = 0.013), respectively. Patients’ weight in the retrocalcaneal partial tear and in the midportion partial tear group was 74 ± 15.5 (range, 47–115) and 83 ± 11.6 (range, 65–110) kg (*p* = 0.046), respectively. BMI for patients in the retrocalcaneal partial tear and in the midportion partial tear group was 24 ± 3.8 (range, 19–36) and 25 ± 3.1 (range, 21–32) kg (p = 0.307), respectively.

### Preoperative history

In the retrocalcaneal and in the midportion partial tear group, the left/right Achilles tendon was affected in 10/11 and 8/8 patients, respectively. Two patients of the retrocalcaneal partial tear group had bilateral involvement with surgery performed during the study period but only the side operated on first was considered for further evaluation, due to our exclusion criteria.

Only one patient in either group was not involved in regular sports. Running activities (21/37), tennis (7/37 patients), and football (3/37 patients) were the predominant preoperative sports (Table 1). Achilles tendon symptoms developed insidiously in 17/21 (81%) and 9/16 (56%) in the retrocalcaneal partial tear and in the midportion partial tear group, respectively (Table 1). No preceding injuries involving the injured Achilles tendon or systemic medical conditions were specified in 11/21 (52%) patients with retrocalcaneal partial tears and 9/16 (56%) with midportion partial tears. Patients in the retrocalcaneal partial tear and in the midportion partial tear group described prodromal symptoms for 44.9 ± 43.8 (range, 6–180) and 47.1 ± 47.2 (range, 2–132) months (*p* = 0.203), respectively. Patients preoperatively underwent different forms of conservative treatment (Table 1). Preoperative MRI correctly detected 6/11 retrocalcaneal impingement partial tears and diagnosed 5/11 patients in the retrocalcaneal partial tear group as suffering from retrocalcaneal bursitis. In the midportion partial tear group, MRI correctly identified 10/11 midportion Achilles tendon partial tears and diagnosed 1/11 ‘cystic posteromedial column’. The period from onset of symptoms to diagnosis was 32.9 ± 45.8 (range, 0–180) and 5.7 ± 4.8 (range, 0–15) months (*p* = 0.001) in the retrocalcaneal partial tear and in the midportion partial tear group, respectively (Table 1). Preoperatively, 16/20 (80%) and 14/16 (88%) in the retrocalcaneal partial tear and in the midportion partial tear group, respectively, complained about Achilles tendon pain while walking (Table 1). Running activities were preoperatively impossible due to the symptoms since 4.0 ± 5.0 (range, 0–14) and 6.6 ± 4.0 (range, 2–15) months (*p* = 0.078) in the retrocalcaneal partial tear and in the midportion partial tear group, respectively (Table 1).

### Intraoperative findings

All retrocalcaneal partial tears were addressed by retrocalcaneal bursa and Haglund resection, debridement, and repair of the partial tears. All midportion partial tears were debrided and repaired side to side. In 5/16 (31%) of those patients, a plantaris tendon augmentation was added. About 14.3 ± 7.8 (range, 5–40) and 24.7 ± 18.7 (range, 5–60)% of the local tendons’ cross-section area were excised in the retrocalcaneal partial tear and midportion partial tear group, respectively (*p* = 0.125; Table 2).

### Complications

No major complications occurred in both groups. In the retrocalcaneal partial tear and in the midportion partial tear group, 5/16 (31%) and 4/19 (21%) of the patients, respectively, suffered from postoperative complications, including two and one deep vein thromboses, respectively. One non-compliant patient resumed jogging already 10 weeks after retrocalcaneal partial tear surgery without permission and had a reinjury, which was conservatively treated. One additional patient complained about continuing postoperative pain following a barefoot walk on the beach at 9 weeks postoperatively. One year postoperatively, a reoperation was proposed, but the patient refused. He scored the lowest VISA-A-G value (15 points) at the final follow-up (Table 2).

Postoperative running activities were resumed 5.2 ± 2.7 (range, 2–13) and 4.6 ± 1.2 (range, 3–7) months in the retrocalcaneal partial tear and in the midportion partial tear group, respectively (*p* = 0.492; Table 2).

### Presentation at last clinical examination

The final clinical investigations were held 10 (range 0–69) and 6.5 (1–23) months postoperatively for the retrocalcaneal partial tear and the midportion partial tear group, respectively (*p* = 0.624). At final clinical investigation, tenderness in the operated Achilles tendon area was still found in 4/18 (22.2%) and 5/16 (31.3%) in the retrocalcaneal partial tear and the midportion partial tear patients, respectively. Due to persisting pain, postoperative MRI investigations were performed in two and one patients of the retrocalcaneal partial tear and the midportion partial tear group, respectively. Ultrasound revealed power Doppler grade 0–II in 14/18 (77.8%) and 13/16 (81.3%) of the patients in the retrocalcaneal partial tear and the midportion partial tear group, respectively, while grade III or IV were found in 4/18 (22.2%) and 3/16 (18.8%). In the midportion partial tear group, Achilles tendon sagittal diameter was 12 ± 3.4 (range, 7–18 mm). Respective values were not recorded for the retrocalcaneal partial tear group.

## Discussion

This study demonstrates that open surgery for Achilles tendon partial tears when recalcitrant to conservative treatment leads to excellent results in more than two thirds of our patients, irrespective of the anatomic location of the injury. Postoperative recovery is slow in both entities. This is underlined by the fact that no statistically relevant improvement could be detected between the preoperative and the 3 month postoperative result. From then, evidence for improvement is provided until 1 year postoperatively, while later, a further improvement of the status could not be verified for either group (Tables 3 and 4).

A minimum important clinical difference of 6.5 VISA-A points was formally established for ‘insertional Achilles tendinopathy’ [28]. The mean between group differences were more than 6.5 points at 3 and 12 months and at final follow-up, indicating a tendency towards better outcomes in patients suffering from midportion Achilles tendon partial tears.

The between group differences in height and weight of the patients are a result of the nearly equal (52% male) sex distribution in the retrocalcaneal partial tear group while in the midportion partial tear group only one out of 16 patients (6%) was female. BMI was statistically not different between groups.

Interestingly, there was no bilateral involvement in the midportion partial tear group, but 3/21 (14%) of the retrocalcaneal partial tear group had bilateral involvement during the study period. Nearly all patients were active in sport, and in most instances, the patient’s history revealed a specific initiating event.

Associated pathologies may play a predisposing role for Achilles tendon partial tears. In 90.9%, foot pain was associated with joint pain at other sites [29]. Correspondingly, in both groups of this study, additional preceding injuries to different parts of the body and systemic medical conditions were frequent. Further research should therefore address the pathogenetic relevance of these comorbidities and its possible influence to the VISA-A scores.

Diagnosis of the described conditions is frequently delayed, ranging from 1 to 180 months, but the midportion Achilles tendon partial tears are diagnosed earlier (median = 5 vs. 21 months, *p* = 0.001). The analysed data cannot explain this difference. The lower chronic status of the injured midportion Achilles tendons, however, may be responsible for the between group difference 12 months postoperatively and at final follow-up. In contrast, a previous study found no statistically relevant difference between the 12-month results of partial tears in the midportion and retrocalcaneal area [30].

The role of local cortisone injections during the preceding conservative treatment of Achilles tendinopathy and retrocalcaneal bursitis is a matter of debate. Systematic research does not support injection therapy in general [31]. ‘Long-term harms to tendon tissue and cells associated with glucocorticoid injections’ are assumed [32], also following injections into the retrocalcaneal bursa [33]. In our retrocalcaneal and midportion partial tear group 13/21 (62%) and 3/16 (19%) of the respective patients had previous cortisone injections.

Literature evaluating partial Achilles tendon tears is rare. It is to assume, that the initial lesion for retrocalcaneal partial tear is impingement resulting from retrocalcaneal bursitis [14, 15, 30, 34].

The strength of this study is that a single orthopaedic surgeon performed all procedures in a standardised manner. Rigorous inclusion and exclusion criteria produced well-defined groups for comparison with the so far longest follow-up. Another strength is the longitudinal design to demonstrate that interval improvement of the injured Achilles tendons at any level is slow and requires about 1 year.

There are inherent limitations to this study. There are low numbers in the groups. Therefore, the tendency towards superiority of the midportion Achilles tendon partial tear group is not robust enough, and larger groups are required to further underline these results. In principle, a selection bias could arise when patients with good results would be more willing to answer the VISA-A-G questionnaire. Consequently, excluding patients with a missing final follow-up could lead to positively overestimating the results. However, there was no statistical difference between the 12 months and final follow-up results between all patients who completed the 12-month questionnaire and those recruited for further calculations (all *p* > 0.195). Finally, relying on a patient related and therefore subjective outcome measure could be criticised. However, the VISA-A is ‘a valid and reliable index of the clinical severity of Achilles tendinopathy’ [22]. It is proposed for ‘clinical measurement studies’ [35]. Since its development, it was cross-culturally translated and adapted to all major languages and is globally accepted [25, 36]. This is important to make results of different researchers internationally comparable. Additionally, it ‘seems suitable for both clinical rating and quantitative research’ [37]. Endoscopic interventions are becoming more and more popular for retrocalcaneal bursitis/Haglund’s syndrome, and good results are reported also when impingement Achilles tendon lesions were addressed merely by retrocalcaneal bursa and Haglund resection [16, 17]. Direct experimental comparison of endoscopic and open retrocalcaneal bursitis and Haglund resection did not reveal an advantage of one technique over the other [38]. Endoscopic repair techniques are not described for midportion and retrocalcaneal Achilles tendon partial tears so far. Therefore, further clinical research should compare open and endoscopic procedures for partial Achilles tendon tears in the midportion and retrocalcaneal area using standardised procedures in a controlled and randomised design.

Rare case reports document successful conservative treatment of Achilles tendon partial tears in the retrocalcaneal and midportion area [6, 12, 39–41]. The current study included only patients who were unresponsive to conservative treatment. Experimental work in a rat model demonstrated that injury severity had a drastic influence on biomechanical characteristics of the Achilles tendons [42]. It can be speculated, that minor partial tears may be more responsive to conservative treatment modalities. Probably, patients with low functional demands respond better to conservative treatment.

Postoperative care may have an influence on outcome. Patients of both groups in this investigation underwent early functional treatment and wore heel lifts in rehabilitation boots for several weeks. In rat experiments, immobilisation reduced function and fatigue resistance of Achilles tendons with partial tears post-injury [42]. Further clinical studies can demonstrate if this association can be transferred to Achilles tendon partial tears in humans.

MRI and diagnostic ultrasound is ‘used to identify or to confirm’ Achilles tendon partial tears and for distinguishing it from complete ruptures and tendinosis [9]. It is recommended for postoperative care [9]. MRI and ultrasonography can confirm the diagnosis but do not consistently detect partial tears of the Achilles tendons. Specifically, the sensitivity of MRI for diagnosing impingement partial tears is not sufficient in this study (Table 1). This finding underlines previous research, demonstrating that ‘Ultrasound and MRI show only moderate correlation with clinical assessment of chronic Achilles tendinopathy’ [43]. Neither for MRI nor for diagnostic ultrasound a grading system exists to evaluate postoperative Achilles tendons. In a recent study ‘intratendinous’ tears were introduced in the differential diagnosis of Achilles tendon disorders. That pathology was ultrasonographically differentiated from partial tears by fibre discontinuity ‘entirely within’ the tendon [3].

## Conclusion

Achilles tendon partial tears can occur in the midportion area and at the level of the retrocalcaneal bursa. In recalcitrant cases, operative treatment is successful in most cases. VISA-A-G questionnaire demonstrated increasing functionality and decreasing symptoms during the first postoperative year, and results do not deteriorate in the long-term.

## Data Availability

The datasets used and analysed during the current study are available from the author on reasonable request.

## Abbreviations

VISA-A-G: Victorian Institute of Sports Assessment–Achilles tendon–German version
